# Excess mortality related to circulatory system diseases and diabetes mellitus among Italian AIDS patients vs. non-AIDS population: a population-based cohort study using the multiple causes-of-death approach

**DOI:** 10.1186/s12879-018-3336-x

**Published:** 2018-08-28

**Authors:** Barbara Suligoi, Saverio Virdone, Martina Taborelli, Luisa Frova, Enrico Grande, Francesco Grippo, Marilena Pappagallo, Vincenza Regine, Lucia Pugliese, Diego Serraino, Antonella Zucchetto

**Affiliations:** 1 Centro Operativo AIDS, Istituto Superiore di Sanità, via Regina Elena 299, 00161 Rome, Italy; 2 Unit of Cancer Epidemiology, Centro di Riferimento Oncologico di Aviano, IRCCS, via Gallini 2, 33081 Aviano, PN Italy; 3 Integrated system for health, social assistance, welfare and justice, Istituto Nazionale di Statistica, viale Liegi 13, 00198 Rome, Italy; 4 Scientific Directorate, Centro di Riferimento Oncologico di Aviano, IRCCS, via Gallini 2, 33081 Aviano, PN Italy

**Keywords:** HIV/AIDS, Circulatory system diseases, Diabetes mellitus, Multiple causes of death, Standardized mortality ratio

## Abstract

**Background:**

Chronic diseases, chiefly cancers and circulatory system diseases (CSDs), have become the leading non-AIDS-related causes of death among HIV-infected people, as in the general population. After our previous report of an excess mortality for several non-AIDS-defining cancers, we now aim to assess whether people with AIDS (PWA) experience also an increased mortality for CSDs and diabetes mellitus (DM), as compared to the non-AIDS general population (non-PWA).

**Methods:**

A nationwide, population-based, retrospective cohort study was conducted including 5285 Italians, aged 15−74 years, who were diagnosed with AIDS between 2006 and 2011. Multiple cause-of-death (MCoD) data, i.e. all conditions reported in death certificates, were retrieved through record-linkage with the National Register of Causes of Death up to 2011. Using MCoD data, sex- and age-standardized mortality ratios (SMRs) with 95% confidence intervals (CIs) were calculated by dividing the observed number of PWA reporting a specific disease among MCoD to the expected number, estimated on the basis of mortality rates (based on MCoD) of non-PWA.

**Results:**

Among 1229 deceased PWA, CSDs were mentioned in 201 (16.4%) certificates and DM in 46 (3.7%) certificates among the various causes of death. These values corresponded to a 13-fold higher mortality related to CSDs (95% CI 10.8–14.4) and DM (95% CI: 9.5–17.4) as compared to 952,019 deceased non-PWA. Among CSDs, statistically significant excess mortality emerged for hypertension (23 deaths, SMR = 6.3, 95% CI: 4.0–9.4), ischemic heart diseases (39 deaths, SMR = 6.1, 95% CI: 4.4–8.4), other forms of heart diseases (88 deaths, SMR = 13.4, 95% CI: 10.8–16.5), and cerebrovascular diseases (42 deaths, SMR = 13.4, 95% CI: 9.7–18.2). The SMRs were particularly elevated among PWA aged < 50 years and those infected through drug injection.

**Conclusions:**

The use of MCoD data disclosed the fairly high mortality excess related to several CSDs and DM among Italian PWA as compared to non-PWA. Study findings also indicate to start preventive strategies for such diseases at a younger age among AIDS patients than in the general population and with focus on drug users.

## Background

The mortality patterns of HIV-infected people have radically changed as a consequence of the prolonged survival due to treatment with highly active antiretroviral therapy (HAART) [1]. In particular, mortality rates for AIDS-related conditions have been decreasing over time, while those due to non-AIDS-related ones have decreased only slightly, or remained unchanged [1–5]. Among non-AIDS-related causes of death, chronic diseases, including cancers and circulatory system diseases (CSDs), are currently the most frequent ones in the HIV-infected population [4, 6, 7]. Nonetheless, neoplasms and CSDs are also the commonest causes of death among the general population of same sex and age [7]; therefore, whether HIV-infected people experience a higher mortality for these diseases than the HIV-negative population and the quantification of such an excess are very difficult to assess.

Our study group has previously published results of an excess mortality for several non-AIDS-defining cancers among the Italian population with AIDS (PWA) [8]. In order to further the investigation of conditions related to PWA mortality, we have put the spotlight on CSDs and diabetes mellitus (DM), as DM is not only one of the main risk factors for CSDs but it is also frequently associated to HAART [9, 10].

Some large cohort studies have shown increased incidence risks of ischemic heart diseases and cerebrovascular accidents, including stroke, among HIV-infected patients as compared to HIV-negative people [11, 12]. Furthermore, cardiovascular events seemed to occur at a younger age among HIV-infected individuals [2, 13, 14]. Despite these evidences of an increased incidence of CSDs, very few studies to date have investigated the issue of mortality for such conditions among HIV-infected people, using different methods [2, 15].

Indeed, comparing mortality data of people with and without HIV or AIDS at a population level is a challenging task, mainly because official statistics generally assign HIV or AIDS as the underlying cause-of-death of these patients, thus hiding the presence of other diseases contributing to the death [4, 7, 16, 17]. In our previous investigation [15], conducted between 1999 and 2006, a laborious revision of death certificate text strings had been necessary to identify DM and some cardiovascular diseases as the single cause-of-death of Italian PWA.

The availability in Italy, since 2006, of multiple cause-of-death (MCoD) data (i.e., all the conditions reported in a death certificate) now allows to investigate the role of all possible conditions leading PWA to death besides the single underlying cause-of-death. Moreover, such an approach offers the opportunity to properly compare the presence of a specific disease between PWA and the general population (overcoming the limitation derived from the assignment of HIV/AIDS as the only cause-of-death of people with HIV/AIDS) [7, 8].

This study, taking advantage of MCoD data, aimed to assess whether Italian PWA reported a higher mortality associated to CSDs and DM, in terms of presence in death certificates of a higher than expected frequency of such diseases, as compared to people without HIV/AIDS (non-PWA) in the general population.

## Methods

In order to verify whether an excess mortality related to CSDs and DM exists among PWA, we conducted a nationwide, population-based, retrospective cohort study. Study methods, have been extensively described in a previous paper focused on non-AIDS defining cancers among Italian PWA diagnosed between 2006 and 2011 [8]. Briefly, this study is part of an investigation on the survival and mortality patterns of Italian PWA, with nationwide coverage [18]. Data derived from (a) the National AIDS Registry (RAIDS), which collects data on all people newly diagnosed with AIDS in Italy according to the 1993 revised European definition [19] (i.e., RAIDS does not include HIV-infected people without AIDS), and (b) the National Register of Causes of Death (RCoD), which collects all death certificates issued in Italy [20] and codes MCoD data, according to the International Classification of Diseases 10th revision, ICD-10 [16]. MCoD data include, for each person deceased in Italy, besides the underlying cause-of-death, all the conditions reported in any field of death certificates coded into ICD-10.

RAIDS and RCoD data were linked together for the period 2006–2011. The record-linkage was carried out in observance of the current laws, given the inclusion of this investigation in the Italian National Statistical Plan, after clearance of the Italian Data Protection Authority. In order to improve linkage effectiveness and to reduce bias, we excluded from this study: (a) the records of PWA living in the provinces of Trento and Bolzano (*n* = 4), because names/surnames were not available in RCoD; (b) the records of PWA who were foreign citizens (*n* = 311), because of the higher frequency of spelling errors in names/surnames and the increased probability of losses at follow-up due to migration (i.e., when they die abroad, neither their death nor their date of last follow-up are reported to RCoD; conversely, when Italian citizens die abroad, their death is always recorded in RCoD). The record-linkage was carried out using a validated semi-automated software application that guarantees anonymity and high sensitivity [21]. After the exclusion of false-positive linked records, the correctly matched records between RAIDS and RCoD were considered the ‘PWA deaths’; whereas, the RCoD records that were not matched with RAIDS were considered the ‘non-PWA deaths’ (i.e., deceased people among the general population without AIDS). Furthermore, in order to avoid the inclusion of HIV-infected people among ‘non-PWA deaths’, also death records reporting any AIDS/HIV-related conditions (i.e., ICD-10 codes B20-B24) were excluded from the ‘non-PWA deaths’ group.

The present analysis was restricted to PWA aged 15–74 years at diagnosis or at death. Furthermore, PWA with less than 1-month interval between AIDS diagnosis and death were excluded (because the day of AIDS diagnosis was often missing and in several cases AIDS was diagnosed after death). The final analysis included 1229 ‘PWA deaths’, occurred among 5285 PWA, and 952,019 ‘non-PWA deaths’.

Sex- and age-standardized mortality ratios (SMRs) [22] were calculated as the ratio between the observed number of ‘PWA deaths’ reporting one specific disease in the death certificate (using MCoD) to the expected number of ‘PWA deaths’, estimated on the basis of (a) sex- and age-specific mortality rates among non-PWA multiplied by (b) the person-years at risk among PWA. (a) Mortality rates for non-PWA were computed as the ratio between the observed number of ‘non-PWA deaths’ reporting one specific condition in the death certificate (using MCoD) and the average resident Italian population of same sex and age in the calendar period (after excluding Trento and Bolzano provinces and foreign citizens), as a proxy of person-years at risk of death of non-PWA. (b) PWA’ person-years at risk of death were calculated from the date of AIDS diagnosis to the date of death, or to December 31st, 2011, whichever came first (PWA who had been diagnosed within 74 years of age but who died thereafter were censored at their 75th birthday). Corresponding 95% confidence intervals (CIs) were computed using the exact Poisson method, which is appropriate for rare events [23]. Nonetheless, diseases for which the total number of observed deaths among PWA was below 10 were not reported in tables.

Cardiac arrest, ICD-10 code I46, was excluded from the analysis on CSDs because it is considered a ‘garbage code’, i.e.*,* a code that should not be indicated in the death certificates as it is just the mode of dying and it is not specifically related to the disease process [16].

## Results

The distribution of 5285 Italian PWA diagnosed during 2006–2011, and of the 1229 PWA who had died as of 31st December 2011, according to main characteristics at AIDS diagnosis is described in Table 1. Briefly, the mean follow-up time was 2.5 years, for a total of 14,180 person-years at risk of death. The proportion of deaths occurred among PWA increased with age at AIDS diagnosis, decreased with years of education, and it was particularly high among PWA infected through injecting drug use (IDU).

**Table 1:** Distribution of 5285 Italian people with AIDS (PWA) and of 1229 PWA who had died between the age of 15 and 74 years, according to selected characteristics at AIDS diagnosis. Italy, 2006-2011

Characteristics at AIDS diagnosis	Total PWA	PWA deaths^a^	
	*N* = 5285	*N* = 1229	
	No. (%)	No.	% of deaths among PWA
Sex			
Male	4215 (79.8)	992	23.6
Female	1070 (20.2)	237	22.1
Age at AIDS diagnosis (years)			
15–39	1566 (29.6)	227	14.5
40–49	2273 (43.0)	527	23.2
50–59	976 (18.5)	298	30.5
60–74	470 (8.9)	177	38.5
Calendar year at AIDS diagnosis			
2006-2007	2110 (39.9)	593	28.3
2008-2009	1780 (33.7)	396	22.3
2010-2011	1395 (26.4)	240	17.3
Residence area^b^			
North	2808 (53.1)	652	23.3
Center	1326 (25.1)	278	21.0
South	1065 (20.2)	273	25.6
Education (years)			
< 6	593 (11.2)	168	28.3
6–8	2216 (41.9)	547	24.7
≥ 9	1601 (30.3)	296	18.5
Unknown	875 (16.6)	218	25.3
HIV transmission mode			
Heterosexual intercourse	2093 (39.6)	446	21.5
Male-to-male sexual intercourse	1316 (24.9)	223	17.0
Injecting drug use	1533 (29.0)	473	30.9
Other/undetermined	343 (6.5)	87	25.7
CD4 (cell count/mm^3^)^b^			
≥ 350	493 (9.3)	97	19.7
200–349	585 (11.1)	161	27.7
50–199	1891 (35.8)	449	23.9
<50	2177 (41.2)	476	21.9

^a^Deceased up to 31st Dec 2011

^b^The sum does not add up to the total because of missing values

Table 2 shows the frequency of death certificates reporting CSDs and DM, according to age class, among PWA and non-PWA, respectively. CSDs and DM were definitely less frequent among PWA rather than among non-PWA: CSDs were reported in 16.4% (201 cases) of death certificates of PWA vs 43.7% of non-PWA, and DM was present in 3.7% (46 cases) vs 11.4%. Conversely, if we had considered only the information on the single underlying cause-of-death, 75.6% (152/201) of PWA with CSDs in the death certificate would have died because of HIV/AIDS alone and, similarly, 71.7% (33/46) of PWA with DM.

**Table 2:** Distribution of 1229 PWA and 952,019 non-PWA, according to selected conditions at death^a^. Italy, 2006-2011

Conditions present at death (ICD-10 codes)	PWA deaths			Non-PWA deaths		
		Age class at death			Age class at death	
	Total	15–49 yrs	50–74 yrs	Total	15–49 yrs	50–74 yrs
	*N* = 1229	*N* = 713	*N* = 516	*N* = 952,019	*N* = 115,199	*N* = 836,820
	N (%)	N (%)	N (%)	N (%)	N (%)	N (%)
Circulatory system diseases (I00–I99)^a,b^	201 (16.4)	85 (11.9)	116 (22.5)	416,141 (43.7)	29,215 (25.4)	386,926 (46.2)
Hypertension (I10–I15)	23 (1.9)	6 (0.8)	17 (3.3)	107,931 (11.3)	3974 (3.4)	103,957 (12.4)
Ischemic heart diseases (I20–I25)	39 (3.2)	9 (1.3)	30 (5.8)	149,319 (15.7)	8476 (7.4)	140,843 (16.8)
Acute myocardial infarction (I21)	17 (1.4)	5 (0.7)	12 (2.3)	75,557 (7.9)	6243 (5.4)	69,314 (8.3)
Chronic ischemic heart disease (I25)	22 (1.8)	4 (0.6)	48 (9.3)	93,719 (9.8)	3254 (2.8)	90,465 (10.8)
Other forms of heart diseases (I30–I52)^b^	88 (7.2)	42 (5.9)	46 (8.9)	175,947 (18.5)	12,902 (11.2)	163,045 (19.5)
Cerebrovascular diseases (I60–I69)	42 (3.4)	*21 (2.9)*	21 (4.1)	95,548 (10.0)	5769 (5.0)	89,779 (10.7)
Stroke (I61, I63–I64)	24 (2.0)	13 (1.8)	11 (2.1)	59,400 (6.2)	3993 (3.5)	55,407 (6.6)
Diabetes mellitus (E10–E14)	46 (3.7)	12 (1.7)	34 (6.6)	108,664 (11.4)	3263 (2.8)	105,401 (12.6)

*PWA*: people with AIDS; *non-PWA* people without AIDS;

^a^Conditions reported in any field of death certificates. Conditions reported in the same death certificate within the same ICD-10 group were counted only once; group of conditions with less than 10 observed cases among total PWA are not shown

^b^But I46 (i.e., cardiac arrest)

In addition, the use of MCoD data allowed to assess the concurrent presence of CSDs and DM at death, which was observed in 21 cases among PWA (1.7% overall, 45.7% out of 46 PWA deaths with DM). These included 7 PWA with both DM and ischemic heart diseases, 5 cases with both DM and hypertension, and 3 cases with both DM and cerebrovascular diseases (data not shown in tables).

Table 3 shows the observed and the expected number of PWA reporting CSDs and DM at death, with the corresponding SMRs, overall and in strata of age. Statistically significant excess risks of death emerged for all the considered diseases. A 12.5-fold excess mortality emerged for CSDs overall (95% CI: 10.8–14.4), including hypertension with a SMR = 6.3 (95% CI: 4.0–9.4); ischemic heart diseases, SMR = 6.1 (95% CI: 4.4–8.4); other forms of heart diseases, SMR = 13.4 (95% CI: 10.8–16.5); and cerebrovascular diseases, SMR = 13.4 (95% CI: 9.7–18.2). DM showed a 13-fold excess risk among PWA as compared to non-PWA (95% CI: 9.5–17.4).

**Table 3:** Observed and expected PWA deaths, with corresponding SMRs, by age at death. Italy, 2006-2011

Conditions present at death^a^ (ICD-10 codes)			Age class at death			
	Total (14,180 person-years)		15–49 yrs. (10,080 person-years)		50–74 yrs. (4100 person-years)	
	Obs./Exp.	SMR (95% CI)	Obs./Exp.	SMR (95% CI)	Obs./Exp.	SMR (95% CI)
Circulatory system diseases (I00–I99)^a,b^	201/16.1	12.5 (10.8–14.4)	85/3.7	22.8 (18.2–28.2)	116/12.3	9.4 (7.8–11.3)
Hypertension (I10–I15)	23/3.7	6.3 (4.0–9.4)	6/0.6	10.3 (3.8–22.4)	17/3.1	5.5 (3.2–8.9)
Ischemic heart diseases (I20–I25)	39/6.4	6.1 (4.4–8.4)	9/1.3	6.9 (3.1–13.1)	30/5.1	5.9 (4.0–8.5)
Acute myocardial infarction (I21)	17/3.7	4.6 (2.7–7.4)	5/1.0	5.22 (1.7–12.2)	12/2.7	4.4 (2.3–7.6)
Chronic ischemic heart disease (I25)	22/3.6	6.1 (3.8–9.3)	4/0.5	7.6 (2.1–19.4)	18/3.1	5.9 (3.5–9.3)
Other forms of heart diseases (I30–I52)^b^	88/6.6	13.4 (10.8–16.5)	42/1.6	27.0 (19.5–36.5)	46/5.0	9.2 (6.7–12.3)
Cerebrovascular diseases (I60–I69)	42/3.1	13.4 (9.7–18.2)	21/0.7	31.8 (19.7–48.6)	21/2.5	8.5 (5.3–13.0)
Stroke (I61, I63–I64)	24/2.0	11.8 (7.6–17.5)	13/0.5	27.9 (14.9–47.8)	11/1.6	7.0 (3.5–12.5)
Diabetes mellitus (E10–E14)	46/3.5	13.0 (9.5–17.4)	12/0.5	26.7 (13.8–46.6)	34/3.1	11.1 (7.7–15.4)

*PWA* people with AIDS, *SMR* sex and age-standardized mortality ratio, *CI* confidence interval; obs./exp.: observed/expected deaths

^a^Conditions reported in any field of death certificates. Conditions reported in the same death certificate within the same ICD-10 group were counted only once; group of conditions with less than 10 observed cases among total PWA are not shown

^b^But I46 (i.e., cardiac arrest)

The SMRs were particularly elevated among PWA who had died at a younger age (Table 3). For instance, among those who died before 50 years of age, the SMR for CSDs was 22.8 (95% CI: 18.2–28.2, 85 cases) and for DM it was 26.7 (95% CI: 13.8–46.6, 12 cases). The same figures among older PWA were 9.4 (95% CI: 7.8–11.3, 116 cases) and 11.1 (95% CI: 7.7–15.4, 34 cases). In particular, the SMR for cerebrovascular events was 31.8 among PWA dying before the age 50 years (95% CI: 19.7–48.6).

Table 4 shows the SMR according to mode of HIV transmission. PWA who were infected through IDU showed particularly high excess mortality for all the considered diseases. For instance, the SMRs were 31.8 (95% CI: 25.2–39.6, 79 cases) for CSDs and 39.8 (95% CI: 22.8–64.7, 16 cases) for DM.

**Table 4:** Observed and expected PWA deaths, with corresponding SMRs, by mode of HIV transmission. Italy, 2006-2011

Conditions present at death^a^ (ICD-10 codes)	Mode of HIV transmission^c^			
	Injecting drug use (473 deaths, 4048 person-years)		Sexual intercourse^d^ (669 deaths, 9302 person-years)	
	Obs./Exp.	SMR (95% CI)	Obs./Exp.	SMR (95% CI)
Circulatory system diseases (I00–I99) ^a,b^	79/2.5	31.8 (25.2–39.6)	108/12.2	8.9 (7.3–10.7)
Hypertension (I10–I15)	6/0.5	12.6 (4.6–27.4)	16/2.9	5.6 (3.2–9.1)
Ischemic heart diseases (I20–I25)	10/1.0	10.5 (5.0–19.3)	27/4.9	5.5 (3.7–8.1)
Acute myocardial infarction (I21)	5/0.7	7.6 (2.5–17.7)	11/2.8	4.0 (2.0–7.1)
Chronic ischemic heart disease (I25)	4/0.4	9.1 (2.5–23.4)	17/2.8	6.0 (3.5–9.6)
Other forms of heart diseases (I30–I52)^b^	34/1.0	33.9 (23.5–47.4)	45/5.0	9.0 (6.6–12.1)
Cerebrovascular diseases (I60–I69)	16/0.4	37.0 (21.2–60.1)	22/2.4	9.2 (5.7–13.9)
Stroke (I61, I63–I64)	8/0.3	26.5 (11.4–52.2)	13/1.5	8.4 (4.5–14.3)
Diabetes mellitus (E10–E14)	16/0.4	39.8 (22.8–64.7)	26/2.8	9.3 (6.1–13.7)

*PWA* people with AIDS, *SMR* sex and age-standardized mortality ratio, *CI* confidence interval; obs./exp.: observed/expected deaths

^a^ Conditions reported in any field of death certificates. Conditions reported in the same death certificate within the same ICD-10 group were counted only once; group of conditions with less than 10 observed cases among total PWA are not shown

^b^But I46 (i.e., cardiac arrest)

^c^The sum does not add up to total PWA because “other HIV transmission modes” were not reported

^d^Including both male-to-male and heterosexual intercourse

## Discussion

This nationwide, population-based study found a fairly high mortality excess among Italian PWA, in terms of presence in death certificates of a higher than expected frequency of several CSDs and DM, as compared to the general population. This excess mortality was particularly high among younger PWA and among those infected through IDU. The use of all causes contributing to death, besides the single underlying cause-of-death, was crucial in order to assess and quantify the excess mortality, because it disclosed the contribution of CSDs and DM among the causes of death of PWA. Nonetheless, the proportions of death certificates reporting either CSDs or DM were relatively small among PWA, and appeared even less relevant when compared with the same proportions among of non-PWA, for whom CSDs and DM are very common causes of death. The use of SMRs allowed to verify that the number of deaths associated with several CSDs and DM among PWA was higher than expected, an information that could not emerge using a comparison between proportions [7].

The magnitude of SMRs was higher than in our previous investigation focused only on DM, myocardial infarction, and chronic ischemic heart diseases [15]; it should be noted, however, that those findings were based on the underlying cause-of-death alone (after a manual revision of death certificates). To our knowledge, there are no other studies estimating SMRs for CSDs or DM among HIV/AIDS patients, as most investigations were focused on incidence and not on mortality [9, 11–14]. A population-based investigation conducted in New York City found an adjusted mortality rate ratio due to cardiovascular diseases of 1.56 (95% CI: 1.49–1.60) among HIV-infected people vs. the general population, using the underlying cause-of-death [2]. The mortality risk was significantly higher in all age groups up to age 65 years [2]. We also found a particularly elevated excess mortality associated with CSDs and DM among younger PWA, as compared to non-PWA. These results are in line with other studies investigating the incidence –not the mortality– of stroke among HIV-positive men [13] and of cardiovascular diseases among HIV-positive women [14], thus indicating that such conditions affect people with HIV/AIDS at a younger age as compared to the HIV-negative population.

Our findings on mortality reflect the increased incidence risk reported for these chronic diseases among HIV-infected people vs. the general population. An Italian retrospective cohort study [11] found a 2-fold higher incidence (standardized incidence ratio, SIR = 2.02) of cardiovascular events, including acute myocardial infarction and stroke, among HIV-infected patients living in Brescia (northern Italy) as compared to the general population. A national study conducted in Denmark [12] also found a 2-fold increased risk of ischemic heart diseases among HIV-infected people after HAART initiation, as compared to a population-based control cohort.

The elevated excess mortality among PWA for CSDs and DM can be related to the higher prevalence of behavioral risk factors among HIV-infected individuals, such as smoking, drugs and alcohol abuse, obesity, unhealthy diet, and of co-morbidities including viral co-infections [2, 3, 6, 24–27]. Unfortunately, information on these risk factors was not systematically available in death certificates. Therefore, we were not able to disentangle the impact on the mortality of PWA due to AIDS itself or to other risk factors. Actually, the previously mentioned risk factors have been shown to have an independent impact on the survival of HIV-infected patients on HAART [28]. The very elevated SMRs we found among PWA who had been infected via IDU, could be mainly attributable to their exposure to drug addiction and alcohol abuse and to co-morbidities, such as hepatitis C virus (HCV) infection [28].

Adverse consequences of HAART can also have an important role in this increased mortality [12]. Indeed, HAART can enhance insulin resistance and alter lipid metabolism and may be associated to metabolic syndrome (i.e., a constellation of cardio-metabolic abnormalities, including glucose intolerance, hyperglycemia, DM, abdominal obesity, hypertension and dyslipidemia) [26]. DM and metabolic syndrome are among the main risk factors for CSDs and a recent meta-analysis [9] reported that people infected with HIV who received HAART had a 4-fold higher odd of developing DM than those HAART naïve. Definitely, the increased survival and the growing aging of individuals receiving HAART have a crucial role in the incidence of and mortality for DM and cardiovascular complications.

Moreover, cardiovascular complications are significantly more frequent among diabetic patients with HIV compared to non-diabetic ones [11, 29]. In the present study, it is worth noting that in 21 PWA, out of 46 (45.7%) with DM and out of 201 (10.4%) with CSDs, both DM and CSDs were mentioned in the death certificate.

We have also checked the concurrent presence of other diseases (different from AIDS-defining ones) together with DM and CSD and we have found that they were rarely present at the same time. For instance, we investigated the co-presence of non-AIDS-defining cancers, as they share common risk factors (e.g., tobacco smoking) with CSDs and DM. We found that, out of 201 PWA death certificates with CSDs, 13 reported also a non-AIDS-defining cancer (i.e., 3 lung, 3 liver, 2 leukemias, 2 Hodgkin’s lymphomas, 1 melanoma, 1 pancreas, 1 larynx), and that, out of 46 PWA death certificates with DM, 3 reported also a non-AIDS-defining cancer (i.e., 1 lung, 1 liver, 1 thyroid).

One of the major study strengths was the use of MCoD, which allowed to identify diseases that, otherwise, would have been hidden by the use of the underlying cause-of-death among people with HIV/AIDS [4, 7, 17]. The recent availability of MCoD data in Italy has allowed to analyze a wide spectrum of conditions classified into ICD-10 codes, thus improving internal and external validity of our study results. Furthermore, the accessibility of MCoD data also for non-PWA allowed us to make a direct comparison between the two groups, given the use of the same coding rules for both groups, thus limiting the potential effect of information bias. Nonetheless, we cannot exclude that the knowledge of HIV infection status could have affected the reporting of various diseases.

Another relevant strength of this study was the completeness of data sources, as both the RAIDS and the RCoD are population-based registries with full coverage of the Italian population. A further strength was the high sensitivity of the record-linkage procedure [21], which allows to amend for spelling errors in names and common errors in dates. It should be noted, however, that our study findings cannot be extended to HIV-infected people without AIDS diagnosis, as they were obtained from the National AIDS Registry, which does not include HIV-only patients. As a consequence, SMRs could be different in the case of HIV-infected people at an earlier stage of immunodeficiency for whom the competitive role of AIDS-defining illnesses at death should be null. Nonetheless, this study methodology could be of aid for the conduction of similar studies among HIV-infected people and for a comparison of the study findings.

## Conclusions

Due to HAART, HIV has become a chronically manageable illness, and our study findings further claim for implementing preventive strategies for chronic diseases, including several CSDs and DM, to reduce not only the incidence but most of all the mortality related to these conditions. Primary preventive actions targeted to smoking and alcohol drinking cessation, healthy diet, weight loss, and physical activity can have an impact in improving the survival of HIV-infected people. Furthermore, risk factors for CSDs should be regularly assessed in this population, together with screening for the diagnosis of DM, and prompting clinicians to shift to lipid/glucose-neutral HAART regimens, depending on individual metabolic response. Finally, as HIV-infected patients with DM are at a particularly high risk of cardiovascular diseases, they should be managed following comprehensive interventions [10]. Specific care and recommendations should focus on individuals younger than 50 years and on those infected through IDU.

## Abbreviations

CI: Confidence interval
CSD: Circulatory system diseases
DM: Diabetes mellitus
ICD-10: International classification of diseases 10th revision
IDU: Injecting drug use
MCoD: Multiple cause-of-death
Non-PWA: People without AIDS
PWA: People with AIDS
RAIDS: National AIDS Registry
RCoD: National Register of Causes of Death
SMR: Standardized mortality ratio
